# Impact of fat on the left atrial roof identified using intracardiac echocardiography during pulmonary vein isolation procedures

**DOI:** 10.1016/j.hroo.2024.11.001

**Published:** 2024-11-09

**Authors:** Yuhi Hasebe, Takashi Noda, Makoto Nakano, Takahiko Chiba, Hiroyuki Sato, Nobuhiko Yamamoto, Tomohiro Ito, Koji Kumagai, Satoshi Yasuda

**Affiliations:** 1 Department of Cardiovascular Medicine, Tohoku University Graduate School of Medicine, Sendai, Japan; 2 First Division of Internal Medicine (Cardiovascular Medicine), Tohoku Medical and Pharmaceutical University Graduate School of Medicine, Sendai, Japan

**Keywords:** Atrial fibrillation, Pulmonary vein isolation, Intracardiac echocardiography, Fat, Septopulmonary bundle

## Abstract

**Background:**

Previous studies have reported the presence of fat between the septopulmonary bundle and the septoatrial bundle on the left atrial (LA) roof. This fat may increase the wall thickness and protect the septopulmonary bundle from radiofrequency energy, potentially leading to conduction gaps.

**Objective:**

This study aimed to determine whether fat on the LA roof can be identified using intracardiac echocardiography (ICE) and whether its presence affects the procedural outcomes of pulmonary vein isolation (PVI).

**Methods:**

We evaluated 94 patients undergoing first-time radiofrequency catheter ablation for atrial fibrillation (60 men [63.8%]; mean age 65.7±10.7 years; 46 with paroxysmal atrial fibrillation [48.9%]) between February 2021 and September 2023. ICE was used to visualize the LA roof, and hypoechoic regions suggestive of fat were marked within the CARTOSOUND map (Biosense Webster, Irvine, CA). PVI was then performed with a personalized isolation line, avoiding fat regions when feasible.

**Results:**

Fat on the LA roof was identified in 35 of 94 patients (37.2%). Conduction gaps on the left pulmonary vein roof were observed in 7 of 35 patients with fat (20.0%) and 1 of 59 patients without fat (1.7%) (*P*=.004). Among patients with conduction gaps, 7 of 8 (87.5%) had a PVI line that crossed a fat region. No significant differences were noted in conduction gaps in other areas between the 2 groups.

**Conclusion:**

The findings indicate that the presence of fat on the LA roof, as identified using ICE, may be associated with a higher incidence of conduction gaps after PVI.

**Key Findings**

▪ Fat between the septoatrial bundle (SAB) and the septopulmonary bundle (SPB) on the left atrial roof could be identified using intracardiac echocardiography.
▪ Fat separating the SAB and SPB was present in 37.2% of patients undergoing pulmonary vein isolation (PVI).
▪ The fat region was associated with conduction gaps after PVI when it extended beyond the PVI line on the left pulmonary vein roof.

## Introduction

Pulmonary vein isolation (PVI) is a widely accepted therapy for patients with symptomatic atrial fibrillation (AF).^1^ While achieving durable PVI is a primary goal, pulmonary vein (PV) reconnections remain a leading cause of AF recurrences.^2^^,^^3^ The left atrium (LA) and the areas surrounding the PVs are anatomically heterogeneous, and the wall thickness (WT) varies among patients.^4^ The ability to create transmural lesions can be limited at certain anatomical sites where the WT is greater, such as the left lateral ridge or the roof of both PVs; however, advances in quantitative ablation techniques have improved the transmurality and continuity of ablation lesions.^5^, ^6^, ^7^

Another important consideration is the presence of distinct and separate epicardial muscle bundles. The LA has a bilayer architecture, with the septopulmonary bundle (SPB) epicardially overlapping the septoatrial bundle (SAB) on the roof and posterior wall.^8^^,^^9^ Recent studies have highlighted the effects of epicardial connections on PVI and roofline ablation outcomes.^10^ It has been proposed that breakthroughs involving the SPB may contribute to epicardial connections.^11^ Pathophysiological reports have shown that fat tissue can be interposed between the SAB and the SPB on the LA roof, potentially increasing the WT and attenuating the radiofrequency (RF) energy, leading to residual connections through the SPB.^12^

Intracardiac echocardiography (ICE) is frequently used to visualize cardiac anatomy during ablation procedures. ICE has been shown to accurately measure the LA-WT, and the use of an ablation index (AI) tailored to the WT has been associated with higher success rates for first-pass isolation.^13^ In this study, we hypothesized that fat tissue between the SAB and the SPB on the LA roof could be identified using ICE and we aimed to investigate whether fat deposition affects the procedural outcomes of PVI.

## Methods

### Study population

This single-center study included patients undergoing first-time radiofrequency catheter ablation (RFCA) for AF at Tohoku University Hospital between February 2021 and September 2023. The protocol was approved by the Institutional Review Board of the Tohoku University School of Medicine (2023-1-1010), and all patients provided written informed consent. The study was conducted in accordance with the ethical standards of the Declaration of Helsinki. The baseline characteristics and laboratory data were collected before RFCA. Transthoracic echocardiography was used to assess the left atrial diameter, left atrial volume index, and left ventricular ejection fraction. The geometry of the LA and PVs was reconstructed using a 320-slice computed tomography (CT) (Aquilion ONE/ViSION Edition, Toshiba Medical Systems, Japan), with patients excluded if they had renal dysfunction or an allergy to iodinated contrast agents. All patients received oral anticoagulant therapy with either a vitamin K antagonist or a direct oral anticoagulant for at least 4 weeks before RFCA, and antiarrhythmic drugs were discontinued 2 days before the procedure. Exclusion criteria included age < 18 years, previous prosthetic mitral valve surgery, severe structural cardiac abnormalities (eg, congenital heart disease), severe left ventricular systolic dysfunction (left ventricular ejection fraction < 25%), and pregnancy.

### RFCA for AF

The RFCA procedure was performed under deep sedation with continuous intravenous infusion of dexmedetomidine hydrochloride and propofol. The esophageal temperature was monitored throughout the procedure. Before the transseptal puncture, a bolus of 3000 IU of unfractionated heparin was administered. The activated clotting time was measured every 15 minutes after the initial heparin dose, and additional heparin boluses were given to maintain an activated clotting time of >300 seconds. A 7-F duodecapolar catheter (BeeAT, Japan Lifeline Co., Ltd., Tokyo, Japan) was placed in the coronary sinus via the right internal jugular vein. An 8-F long sheath (SL0, Abbott, St. Paul, MN) and an 8.5-F deflectable sheath (Agilis, Abbott) were introduced into the LA through a transseptal puncture, which was guided by an 8-F ICE catheter (SoundStar, Biosense Webster, Diamond Bar, CA). Mapping was conducted using either a ring-shaped decapolar catheter (LASSO; Biosense Webster) or a multielectrode catheter (PENTARAY, Biosense Webster). The CARTO 3 system (Biosense Webster) was used to integrate the voltage map with the LA image reconstructed from cardiac CT.

### Fat assessment with ICE

**Figure 1:**
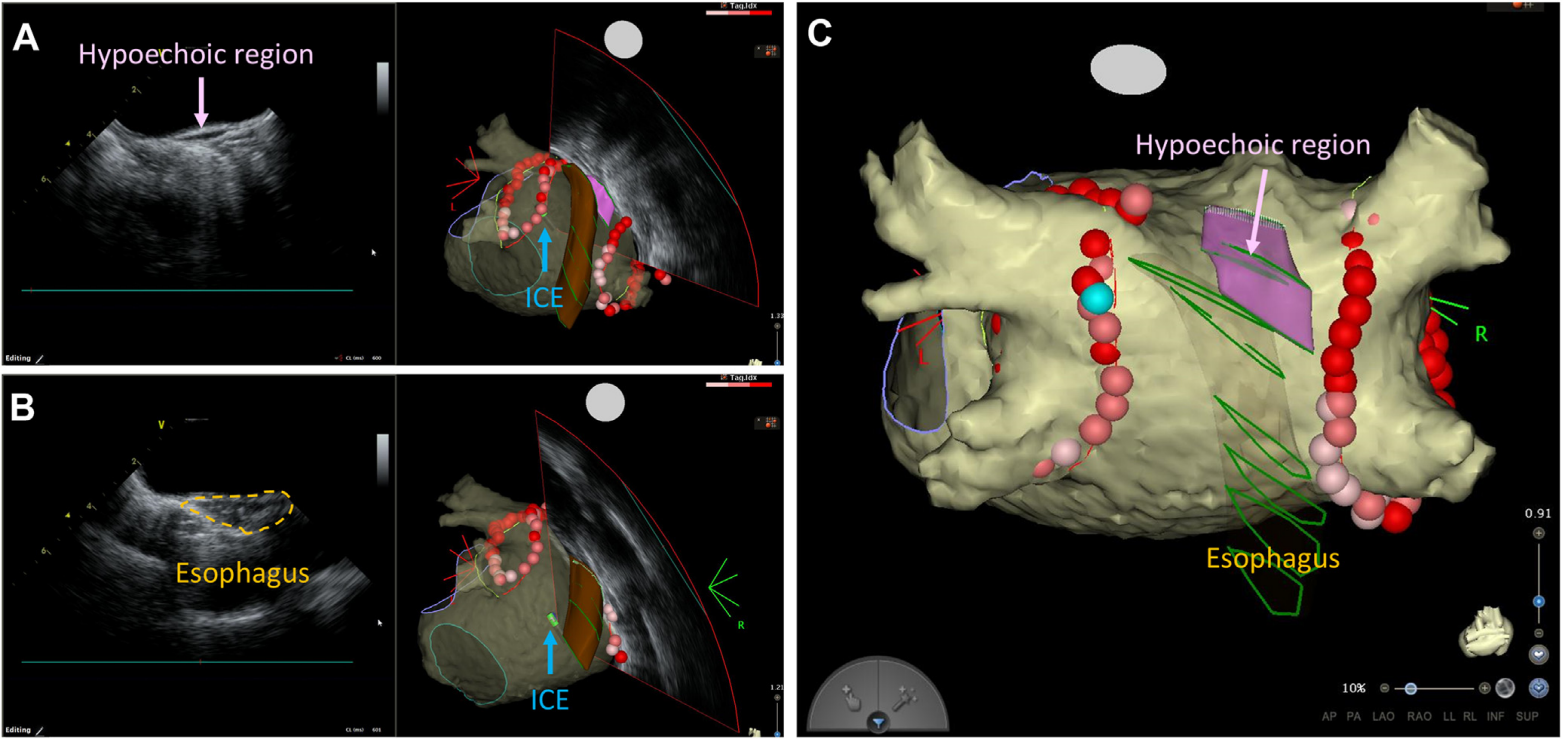
Visualization of hypoechoic regions on the LA roof using ICE. **A:** ICE image showing a hypoechoic region on the LA roof. The hypoechoic region is highlighted in *pink* on the CARTOSOUND map. **B:** ICE image depicting the esophagus on the LA posterior wall, marked in *brown* on the CARTOSOUND map. **C:** CARTOMerge image of the LA, showing the hypoechoic region suggestive of fat and the esophagus as identified using ICE. ICE = intracardiac echocardiography; LA = left atrium/atrial.

ICE imaging of the LA was performed before PVI. The ICE catheter (SoundStar) was advanced into the LA via an Agilis sheath, with its tip carefully positioned at the center of the LA under fluoroscopic guidance. The catheter was gently rotated without deflection to obtain images perpendicular to the posterior wall of the LA. The esophagus was manually outlined on the posterior wall segments. ICE images were captured during the diastolic phase. The catheter was then rotated clockwise to capture images perpendicular to the LA roof. When a hypoechoic region suggestive of fat was identified beyond the endocardial boundary, it was manually outlined and incorporated into the CARTOSOUND map (Figure 1). To validate the accuracy and reliability of the fat assessment, ICE findings were compared with those of CT scans in the same patients. Fat regions were identified on CT by referencing the attenuation values (−50 to −100 Hounsfield unit). The validation analysis was not performed in patients with low-resolution CT imaging due to the high rate of AF or plain CT due to renal dysfunction.

### PVI and other ablation

PVI was performed using a point-by-point wide-area circumferential ablation pattern with an open-irrigated contact-force sensing catheter (ThermoCool SmartTouch SF, Biosense Webster). When a hypoechoic region was not identified using ICE, the wide-area circumferential ablation lines were created >10 mm outside the PV ostia, targeting areas where the local electrograms exhibited no near-field PV signals. For the left pulmonary veins (LPVs), ablation was performed just inside the ridge anterior to the veins. Ablation on the inter-PV carina was avoided as part of the initial lesion set unless necessary to achieve complete PVI. When a hypoechoic region was identified using ICE, the ablation line was adjusted to avoid crossing it. When a hypoechoic region ranged ≥10 mm on the inner side of the PV antrum, an ablation line crossing fat was allowed to decrease the risk of PV stenosis.

RF energy was delivered in the power-controlled mode at 30–40 W, with an irrigation flow rate of 8 mL/min for ≤30 W and 15 mL/min for >30 W. Lesions were guided by the AI targets: 450 on the roof and anterior walls and 400 on the posterior and inferior walls, with an interlesion distance of 4 mm. In areas where the esophagus was detected using ICE, RF energy was delivered at 40 W for ≤5 seconds on the posterior and inferior segments.

If AF persisted after PVI, electrical cardioversion was performed to restore sinus rhythm. *First-pass isolation success* was defined as electrical isolation of the ipsilateral PVs after completing the initial encircling RF lesion set. If residual PV potentials were observed after the initial lesion set, conduction gaps were assessed with a local activation time map and Ripple map created using the PENTARAY catheter. After assessing conduction gaps, additional RF ablation was performed to eliminate them. When the residual positive electrogram amplitude was observed on the initial line, we applied the additional RF applications there. If it was ineffective, an additional application was allowed inside the initial PVI line. In cases where spontaneous PV reconnections occurred after a waiting period of >20 minutes, conduction gaps were assessed in the same manner. Then, additional RF applications were administered. The analysis of conduction gaps assessed the total number of acute gaps after the initial encircling lesion set and the reconnection gaps after the waiting period.

### Statistical analysis

Normally distributed continuous data are presented as mean ± SD and were compared using the Student *t* test. Categorical data are presented as count (percentage) and were compared using the χ^2^ test or Fisher exact test, when appropriate. A *P* value of <.05 was considered statistically significant. All analyses were performed using JMP 14 software (SAS Institute Inc, Cary, NC).

## Results

### Patient characteristics

The baseline characteristics are summarized in Table 1. Of the 94 patients included, 60 were men (63.8%), with a mean age of 65.7±10.7 years. AF was paroxysmal in 46 patients (48.9%). There were no significant differences in comorbidities between the fat and nonfat groups, and the average CHA_2_DS_2_-VASc score was 2.8±1.5. The LA diameter averaged 40.1±6.6 mm, with no significant differences observed in the transthoracic echocardiography parameters.

**Table 1:** Baseline characteristics

Characteristic	Total (N=94)	Nonfat (n=59)	Fat (n=35)	*P*
				
Age (y)	65.7±10.7	65.1±11.1	66.7±9.9	.49
Male sex	60 (63.8)	35 (59.3)	25 (71.4)	.27
Body mass index (kg/m^2^)	24.5±3.4	24.9±3.5	23.7±3.1	.08
Paroxysmal AF	46 (48.9)	26 (44.1)	20 (57.1)	.29
Hypertension	54 (57.5)	34 (57.6)	20 (57.1)	>.99
Diabetes mellitus	20 (21.3)	14 (23.7)	6 (17.1)	.60
Congestive heart failure	27 (28.7)	20 (33.9)	7 (20.0)	.17
Stroke	12 (12.8)	9 (15.3)	3 (8.6)	.53
Cardiovascular disease	10 (10.6)	5 (8.5)	5 (14.3)	.49
CHA_2_DS_2_-VASc score	2.8±1.5	2.9±1.4	2.6±1.5	.39
eGFR (mL/(min·1.73 m^2^))	64.2±16.0	62.8±15.4	66.6±17.0	.18
BNP (pg/mL)	42.8 (21.9–104.2)	57.2 (24.8–104.8)	37.4 (17.2.6–90.4)	.27
Transthoracic echocardiography				
LA diameter (mm)	40.1±6.6	40.2±6.9	40.0±6.1	.86
LA volume index (2D)	40.1±11.9	39.5±11.5	41.2±12.6	.53
LVEF (%)	60.2±11.5	60.5±11.4	59.7±11.6	.74
E/eʹ	10.5±3.8	11.0±3.5	9.6±4.2	.17

2D = 2-dimensional; AF = atrial fibrillation; BNP = brain natriuretic peptide; E/eʹ = mitral inflow E wave/early diastolic mitral annular velocity; eGFR = estimated glomerular filtration rate; LA = left atrial; LVEF = left ventricular ejection fraction.

Values are presented as mean ± SD, median (interquartile range), or n (%).

### Assessment of the LA roof and posterior wall with ICE

**Figure 2:**
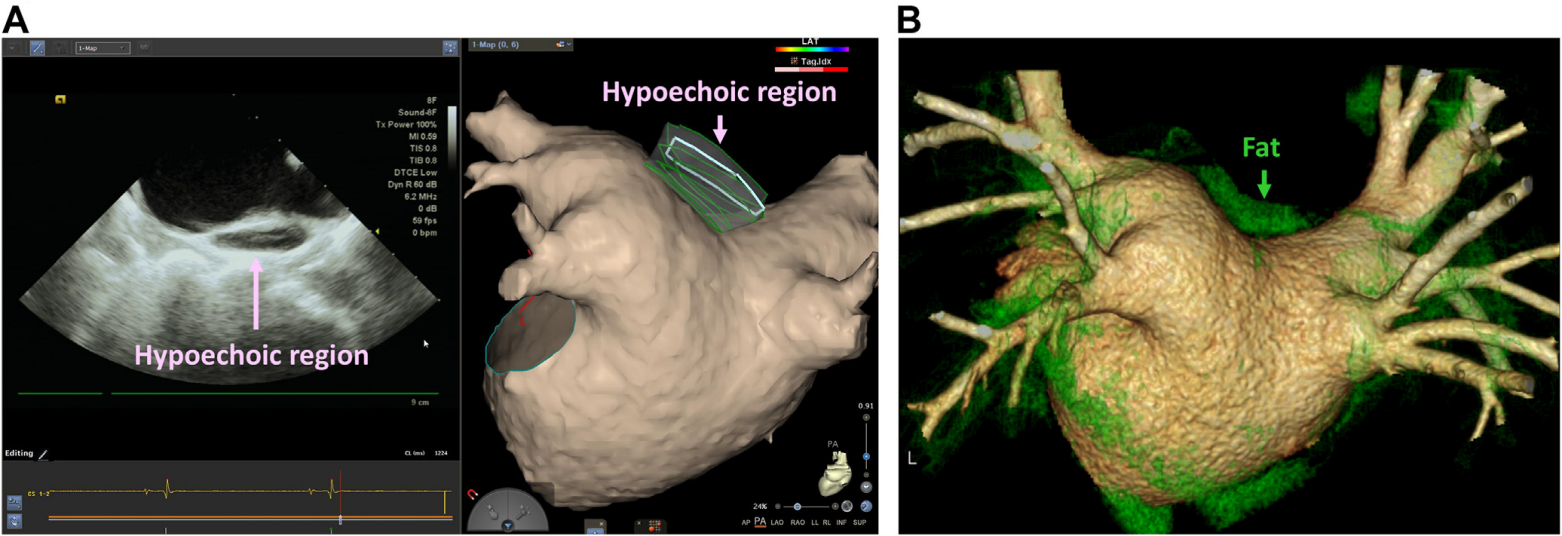
Validation with cardiac CT. **A:** ICE image showing a hypoechoic region on the LA roof. **B:** Cardiac CT image revealing fat around the LA. The fat region, with CT values of −50 to −100 Hounsfield unit, is highlighted in *green*. The hypoechoic region identified using ICE corresponds to the fat region reconstructed using cardiac CT. CT = computed tomography; ICE = intracardiac echocardiography; LA = left atrium/atrial.

**Figure 3:**
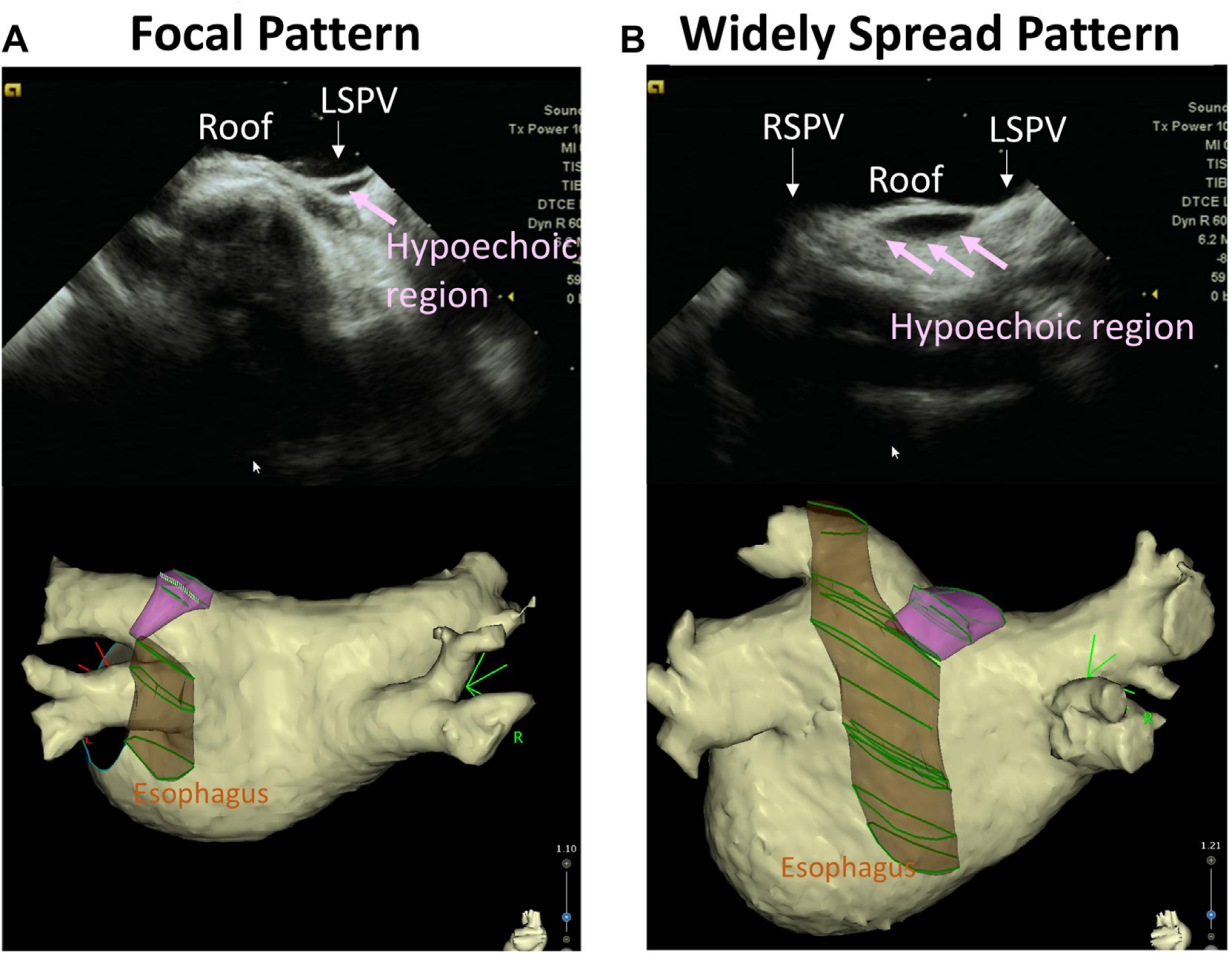
Distribution patterns of fat regions on the LA roof. **A:** Focal pattern of fat at the LSPV-LA junction (18 of 35 [51.4%]). **B:** Widely spread pattern of fat between the LSPV and the RSPV (17 of 35 [48.6%]). LA = left atrium/atrial; LSPV = left superior pulmonary vein; RSPV = right superior pulmonary vein.

The hypoechoic regions on the LA roof, suggestive of fat, were identified using ICE in 35 of 94 patients (37.2%) (fat group). These hypoechoic regions were consistent with the fat regions identified on cardiac CT in 32 patients (91.4%) (Figure 2). Three patients with low-resolution CT imaging due to the high rate of AF or plain CT due to renal dysfunction were excluded from the validation analysis. Two distribution patterns of the fat regions were observed. We classified the distribution patterns on the basis of the extent as follows: a focal pattern at the left superior pulmonary vein (LSPV)–LA junction (18 of 35 [51.4%]) and a widely spread pattern extending between the LSPV and the right superior PV (17 of 35 [48.6%]) (Figure 3).

### PVI and the distribution of conduction gaps

**Figure 4:**
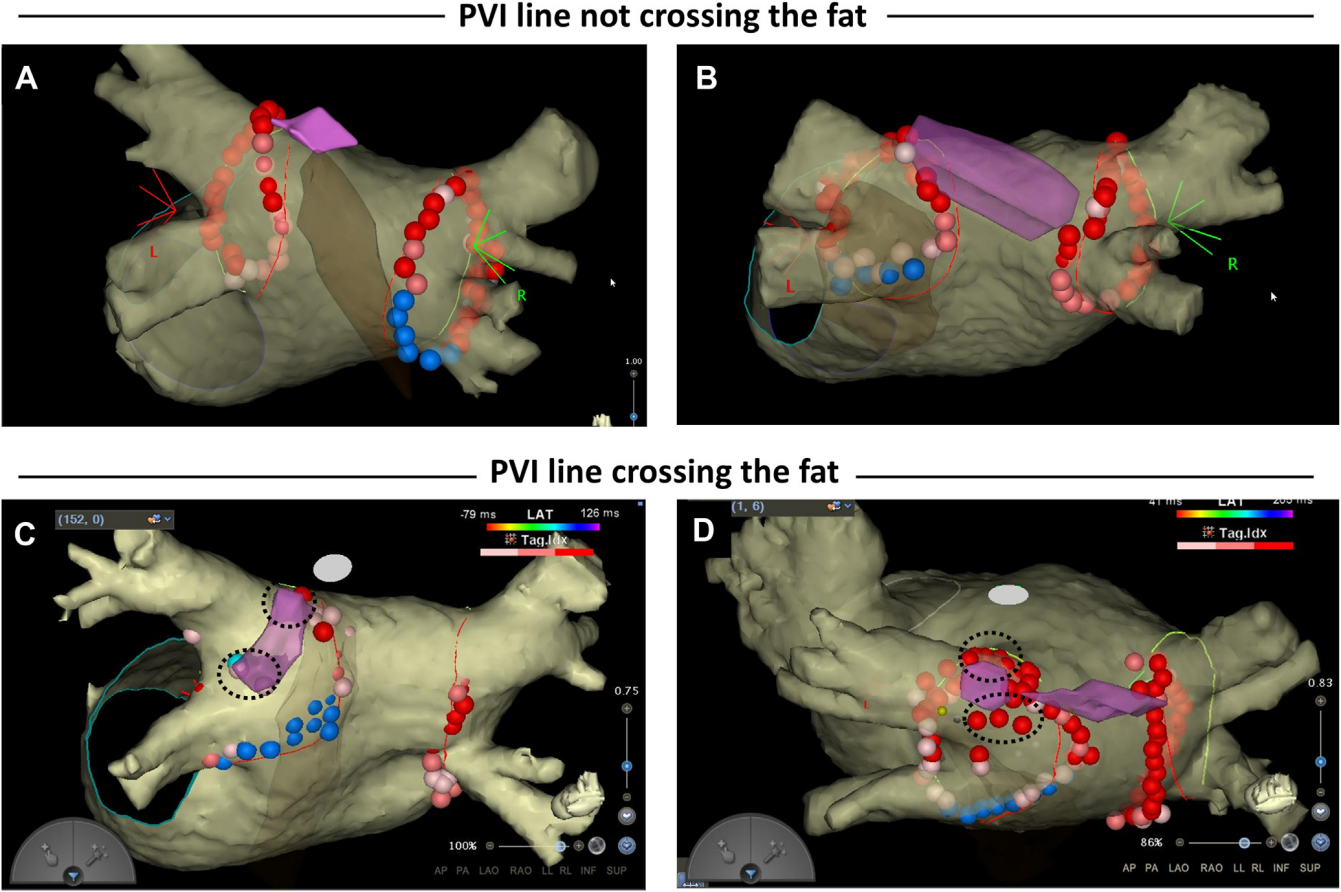
Representative PVI line in patients with fat on the LA roof. **A:** Patient with a focal fat region at the LSPV-LA junction. A modified PVI line that avoided fat achieved first-pass isolation without acute recurrence. **B:** Patient with a widely spread fat region between the LSPV and the RSPV. A modified PVI line avoiding fat was successfully created, resulting in first-pass isolation with no acute recurrence. **C:** Patient with a focal fat region extending from the LSPV-LA junction to the posterior carina. A spontaneous reconnection occurred after first-pass isolation. Reisolation was achieved by ablating the roof and posterior carina adjacent to the fat region (*dotted circle*). **D:** Patient with a widely spread fat region on the inner side of the LSPV. First-pass isolation was not achieved, requiring ablation of the inner side of the PV in front of and behind the fat region (*dotted circle*). The *pink area* represents the fat region identified using ICE. The *brown area* indicates the esophagus identified using ICE. ICE = intracardiac echocardiography; LA = left atrium/atrial; LSPV = left superior pulmonary vein; PV = pulmonary vein; PVI = pulmonary vein isolation; RSPV = right superior pulmonary vein.

**Figure 5:**
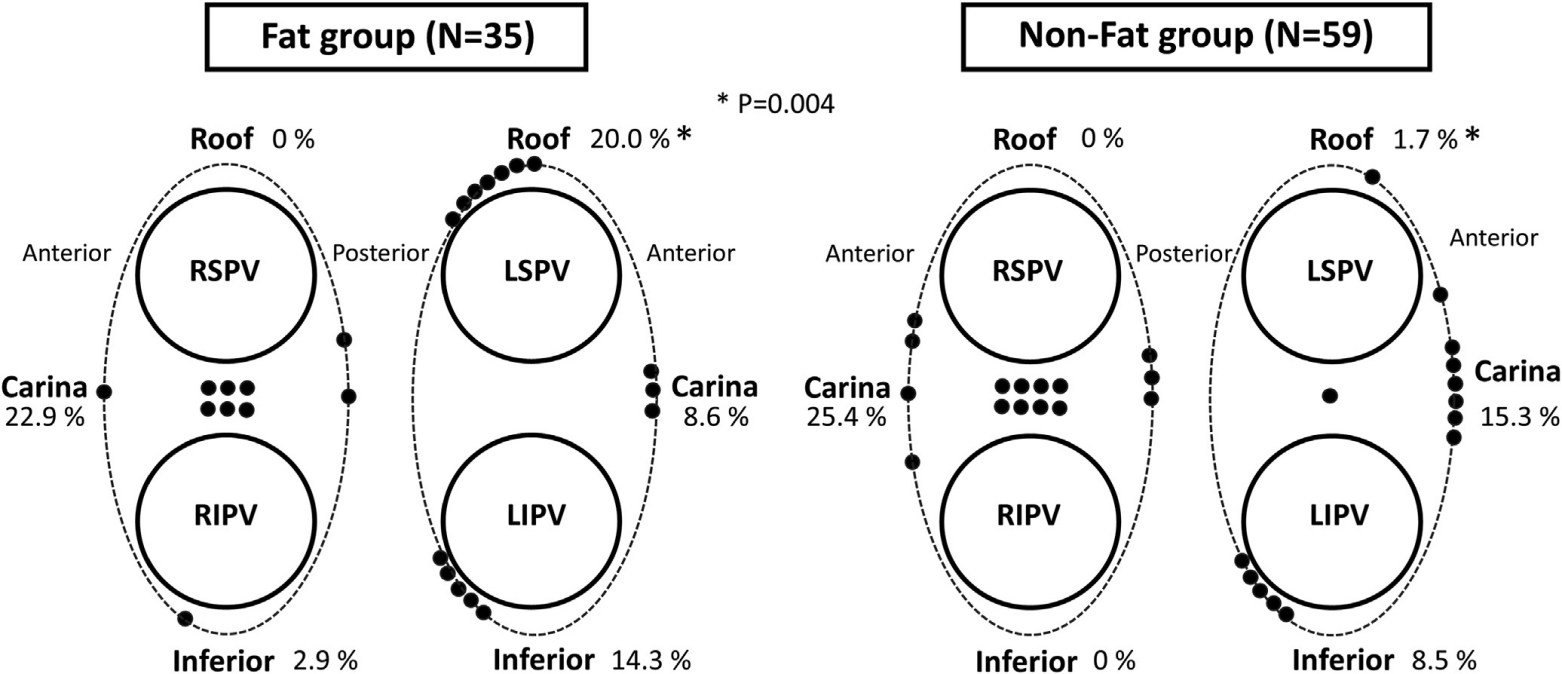
Distribution of conduction gaps. Diagram showing the percentage of segments with conduction gaps in the fat group (*left*) and the nonfat group (*right*). The *black dots* represent the sites of conduction gaps observed after the initial PVI set and spontaneous reconnections. LIPV = left inferior pulmonary vein; LSPV = left superior pulmonary vein; PVI = pulmonary vein isolation; RIPV = right inferior pulmonary vein; RSPV = right superior pulmonary vein.

First-pass isolation was achieved in 88.3% of LPVs and 79.8% of right pulmonary veins (RPVs) across all patients. In the fat group, the success rate of first-pass isolation for LPVs was 82.9% (29 of 35) as compared with 91.5% (54 of 59) in the nonfat group (*P*=.32). In 27 of 35 patients (77.1%) in the fat group, the PVI line did not cross the fat region at the LSPV-LA junction. This included cases where the PVI line had to be adjusted to avoid the fat region on the left posterior roof (Figures 4A and 4B). Patients in the fat group with a PVI line that did not cross the fat region had a higher success rate of first-pass isolation for LPVs than did those whose PVI line crossed the fat region (96.3% [26 of 27] vs 37.5% [3 of 8]; *P*=.0009). Conduction gaps after the initial lesion set were observed on the LPV roof in 4 of 8 patients with a PVI line that crossed the fat region. After a period of observation, reconnections on the LPV roof were observed in 3 other patients in this subgroup. In total, acute or late conduction gaps on the LPV roof were observed in 7 of 8 patients (87.5%) in this subgroup. The distribution of the segments with acute conduction gaps and reconnections is shown in Figure 5. A conduction gap on the LPV roof was observed in 7 of 35 patients (20.0%) in the fat group as compared with 1 of 59 patients (1.7%) in the nonfat group (*P*=.004). In patients from the fat group with conduction gaps on the LPV roof, the additional RF applications on the initial PVI line were ineffective and those gaps were successfully ablated adjacent to the fat region of the inner PVI line (Figures 4C and 4D and Online Supplemental Movie). The RPV carina and posteroinferior wall of the LPV were other common sites where conduction gaps were observed. There was no significant difference in conduction gaps except for the LPV roof between the 2 groups (Figure 5). The procedural time for PVI was 64.3±29.4 minutes in the fat group and 59.0±19.8 minutes in the nonfat group (*P*=.29). The energy requirements for PVI were 57,268±9969 J in the fat group and 56,467±10,875 J in the nonfat group (*P*=.72).

## Discussion

The main findings of this study were as follows: (1) fat was present between the SAB and the SPB on the LA roof in 37.2% of patients who underwent PVI; (2) the fat regions exhibited 2 distribution patterns: a widely spread pattern between the superior PVs and a focal pattern at the LSPV-LA junction; and (3) conduction gaps were observed when the fat region extended beyond the PVI line on the LPV roof.

### Fat between the bilayer architecture of the LA roof

Papez et al first reported that the LA comprises distinct bundles, including the SAB and SPB.^8^ The SAB originates from an interatrial raphe and extends through the body of the LA, while the SPB emerges from the interatrial groove and runs through the anterior wall, roof, and posterior wall, encircling both PVs. The SPB epicardially overlaps with the SAB, forming a bilayer architecture on the LA roof and posterior wall. This bilayer structure has been confirmed by histological studies and diffusion tensor magnetic resonance imaging techniques.^4^^,^^14^ Pambrun et al^12^ histologically described the separation of the SAB and SPB by fat tissue. However, their study, which examined only 5 human donor hearts, did not establish the prevalence of fat tissue infiltration.

Epicardial fat is typically identified as a relatively echo-free space between the myocardium and the pericardium on echocardiography.^15^ ICE offers a higher spatial resolution (0.2–0.3 mm) than does cardiac CT (0.4 mm) and provides real-time anatomical imaging during RFCA.^16^^,^^17^ In this study, hypoechoic regions between the endocardial and epicardial muscular layers identified using ICE were consistent with the low attenuation coefficient of fat (−50 to −100 Hounsfield unit) detected using CT. Our study confirmed the presence of fat separating the SAB and SPB in a significant proportion of patients (35 of 94 [37.2%]). In addition, we observed 2 distribution patterns of the fat region: a focal pattern at the LSPV-LA junction (18 of 35 [51.4%]) and a widely spread pattern between the superior PVs (17 of 35 [48.6%]) (Figure 3). This finding suggests that ICE is a simple and accurate method for identifying mid-layer fat regions on the LA roof.

### Conduction gaps on the LSPV roof after PVI

The CLOSE protocol PVI, using the CARTO VISITAG Module with the AI (Biosense Webster), has been associated with a specificity of 93% for predicting durable ablation segments.^18^ The PRAISE study,^19^ which used the CLOSE protocol PVI for persistent AF, found that PVI durability was achieved in 78% of patients, with late reconnections most frequently occurring in the left anterior/roof regions. Motoike et al^13^ demonstrated that an AI adjustment protocol based on the WT using ICE achieved durable PVI with lower RF requirements as compared with the CLOSE protocol. In their study, the WT of the LSPV roof region was relatively thick (mean 5.1±1.3 mm), with a recommended target AI of 451, which aligns with our target AI setup of 450 for the roof.

In our study, the success rate of first-pass isolation was 88.3% for LPVs and 79.8% for RPVs, consistent with previous reports. We were able to avoid crossing the fat region with the PVI line in 77.1% of patients in the fat group. The success rate of first-pass isolation in patients with a PVI line not crossing the fat region was comparable to that in those without fat. This suggested that a target AI of 450 was effective when creating an optimal PVI line that avoids fat regions at the LSPV-LA junction. Conversely, patients whose PVI line crossed the fat region had a significantly lower first-pass isolation success rate (37.5%). As illustrated in Figure 5, the fat region on the LSPV roof appears to be associated with conduction gaps, which may complicate achieving first-pass isolation. When PVI lines cross the fat region, we might need to set a higher target AI than 450 within a safe range below the fat regions.

It has been shown that epicardial fat represents a major limitation for achieving adequate lesions in epicardial RFCA. The pathophysiological mechanism is hypothesized that the low electrical and thermal conductivity of fat may make it less likely for RF current and heat conduction, respectively, to penetrate into the underlying myocardial tissue.^20^ In the present study, these pathophysiological characteristics of the mid-layer fat might have acted as an effective barrier for the SPB against endocardial RF ablation, resulting in an inadequate lesion formation.

### Study limitations

Several limitations of this study should be noted. First, it was a single-center study where all fat assessments with ICE and RFCA procedures were conducted by a single operator. This design limits the ability to account for the learning curve effect in visualizing fat regions with ICE and in creating personalized PVI lines that avoid these regions. Second, the study lacked a control group to directly compare the effectiveness of the personalized PVI strategy with that of standard ablation techniques. The randomized controlled trial comparing the procedural outcomes of personalized PVI avoiding fat regions with those using conventional ablation lines without fat information would provide more precise insight into the impact of fat on the LA roof. Third, long-term data on the durability of PVI were not available. Fourth, we focused on interposed fat between the SAB and the SPB. Because we did not analyze the overall amount of epicardial fat, it was uncertain whether the AF pattern was related to the extent of atrial cardiomyopathy causing the epicardial fat deposition.

## Conclusion

In this study, we successfully identified the fat regions between the SAB and the SPB using ICE. When fat extends beyond the PVI line, it may contribute to conduction gaps. Therefore, analyzing the LA roof with ICE can be valuable for identifying patients who may face challenges in achieving successful PVI.

## Disclosures

The authors declare that there is no conflict of interest.
