# Supplementary material for: Impact of fat on the left atrial roof identified using intracardiac echocardiography during pulmonary vein isolation procedures
Source: Heart Rhythm O2. 2024 Nov 9;6(1):3–10. doi: 10.1016/j.hroo.2024.11.001 (PMC11993803; doi:10.1016/j.hroo.2024.11.001)
Supplement: Supplemental Material [file mmc2.docx]

**Supplementary Material Legends**

**Supplementary Movie**

Shows a reconnection of the LPV following first-pass isolation in a patient with persistent AF (refer to **Figure 4C**). A ripple map, obtained during pacing from an electrode in the coronary sinus, demonstrates propagation from the LPV roof to the posterior carina across the fat region.
